# Incorporating a real-time automatic alerting system based on electronic medical records could improve rapid response systems: a retrospective cohort study

**DOI:** 10.1186/s13049-021-00979-y

**Published:** 2021-12-04

**Authors:** Seung-Hun You, Sun-Young Jung, Hyun Joo Lee, Sulhee Kim, Eunjin Yang, SAVER team

**Affiliations:** 1 Department of Global Innovative Drugs, Graduate School of Chung-Ang University, 84 Heukseok-ro, Dongjak-gu, Seoul, 06974 South Korea; 2 College of Pharmacy, Chung-Ang University, 84 Heukseok-ro, Dongjak-gu, Seoul, 06974 South Korea; 3 Department of Thoracic and Cardiovascular Surgery, Seoul National University Hospital, Seoul National University College of Medicine, 101 Daehak-ro, Jongno-gu, Seoul, 03080 South Korea; 4 Department of Critical Care Medicine, Seoul National University Hospital, 101 Daehak-ro, Jongno-gu, Seoul, 03080 South Korea; 5 College of Nursing and Mo-Im Kim Nursing Research Institute, Yonsei University, 50-1 Yonsei-ro, Seodaemun-gu, Seoul, 03722 South Korea

**Keywords:** Rapid response team, Clinical alarms, Quality improvements, Resuscitation, Intensive care units

## Abstract

**Background:**

Rapid response systems (RRSs) are essential components of patient safety systems; however, limited evidence exists regarding their effectiveness and optimal structures. We aimed to assess the activation patterns and outcomes of RRS implementation with/without a real-time automatic alerting system (AAS) based on electronic medical records (EMRs).

**Methods:**

We retrospectively analyzed clinical data of patients for whom the RRS was activated in the surgical wards of a tertiary university hospital. We compared the code rate, in-hospital mortality, unplanned intensive care unit (ICU) admission, and other clinical outcomes before and after applying RRS and AAS as follows: pre-RRS (January 2013–July 2015), RRS without AAS (August 2015–November 2016), and RRS with AAS (December 2016–December 2017).

**Results:**

In-hospital mortality per 1000 admissions decreased from 15.1 to 12.9 after RRS implementation (*p* < 0.001). RRS activation per 1000 admissions increased from 14.4 to 26.3 after AAS implementation. The severity of patients’ condition calculated using the modified early warning score increased from 2.5 (± 2.1) in the RRS without AAS to 3.6 (± 2.1) (*p* < 0.001) in the RRS with AAS. The total and preventable code rates and in-hospital mortality rates were comparable between the RRS implementation periods without/with AAS. ICU duration and mortality results improved in patients with RRS activation and unplanned ICU admission. The data of RRS non-activated group remained unaltered during the study.

**Conclusions:**

Real-time AAS based on EMRs might help identify unstable patients. Early detection and intervention with RRS may improve patient outcomes.

**Supplementary Information:**

The online version contains supplementary material available at 10.1186/s13049-021-00979-y.

## Background

Rapid response systems (RRSs) have been developed for patient safety and health care quality improvement [1]. An RRS is different from traditional code team-based systems, as it identifies deteriorating patients in the early phase before the development of collapse and cardiac arrest and is accepted as an essential component of patient safety systems although limited evidence exists regarding its effectiveness [2–5]. RRSs started being used in Korea in 2008, and many centers have recently adopted RRSs owing to the pilot insurance coverage program that started in May 2019 [6, 7].

An RRS comprises an afferent limb, efferent limb, patient safety/quality improvement component with feedback, and administrative structure supporting ongoing training [8, 9]; however, there is no universal consensus regarding the criteria of activations or optimal structure for RRSs [1]. Since operating an RRS requires extensive medical resources, including physicians and well-trained nurses, each hospital has developed a specialized RRS appropriate for its clinical requirements and conditions; such specialized RRSs may be tailored to the composition of RRS members and their working schedules and may include a wide range of activities. Though considerable efforts have been made to recognize a deteriorating patient in the early phase through the RRS, this system depends on manual alerts by ward’s physicians and, thus, may not assure prompt recognition of patients with activation criteria [10, 11]. Hillman et al. reported that only 30% of patients who fulfilled the activation criteria were identified through a manual alert (phone call) [11]. Hence, there have been attempts to develop an early electronic alerting system using variable clinical information including vital signs and laboratory findings in electronic medical records (EMRs) [12–15].

An automatic alerting system (AAS) based on EMRs is convenient for real-time use and may improve the timeliness of RRS. However, several technical and operational challenges exist in adopting a pilot alert monitoring system based on EMRs including EMR scalability, algorithm adaptation, delays of data acquisition, system dissemination, and alert fatigue [12]. Moreover, there is limited evidence regarding the use of AAS compared with conventional primary calling system in terms of activation patterns and clinical outcomes [16]. Although a randomized trial reported a modest improvement on length of hospital stay associated with real-time AAS, improvements in the intensive care unit (ICU) transfer rate or hospital mortality was not observed [13].

Thus, this study aimed to analyze the activation patterns and real-world clinical outcomes of RRS before and after adopting a real-time AAS based on EMRs.

## Methods

### Data source and study population

The RRS has been operational in the surgical wards of Seoul National University Hospital since August 2015, and its use has been extended to cover the medical wards and other clinical laboratory departments. We started using a conventional RRS, triggered by the manual activation of a doctor’s or nurse’s phone call. With manual bedside call activations, we could also perform active screening by medical emergency team (MET) members for high-risk patients, such as those in post-ICU phases and patients with high-risk comorbidities, to identify deteriorating patients earlier. Surgical patients were the first candidates owing to a shortage of available physicians during the daytime. A real-time AAS based on EMRs has been operational with new EMR system since November 2016. We retrospectively analyzed the clinical data of patients in the surgical wards during three periods: pre-RRS (from January 2013 to July 2015, 31 months), RRS without AAS (from August 2015 to November 2016, 16 months), and RRS with AAS (from December 2016 to December 2017, 13 months). All surgical departments (i.e., general surgery, urology, orthopedics, otolaryngology, plastic surgery, neurosurgery, ophthalmology, obstetrics, and gynecology; 692 beds in 21 wards), except those of thoracic and cardiovascular surgery, were included in the analysis.

The study was performed according to the tenets of the Helsinki Declaration and was approved by the Institutional Review Board of Seoul National University Hospital (H-1904-011-1023). The requirement for informed consent was waived.

### Operation of RRS

The RRS was serviced by multidisciplinary ICU intensivists (e.g., anesthesiology, cardiology, general surgery, neurology, neurosurgery, pulmonology, and thoracic and cardiovascular surgery) and by well-trained MET nurses specializing in intensive care. The intensivists worked from 7 AM to 7 PM during weekdays, and the nurses worked from 7 AM to 10 PM in two shifts. The MET was managed separately and not by the traditional code team, and in cases of code events, MET nurses supported code team’s resuscitation.

The conventional RRS input system, i.e., afferent limb, comprised two methods: first was the bedside clinicians’ call for help wherein anyone in the wards could call the MET nurses and second was the active manual screening and proactive rounding of high-risk patients by MET nurses at the candidate wards. Activation criteria and active surveillance criteria of the RRS are shown in Table 1. If the RRS was activated, MET nurses reviewed the indicated patient’s EMR and visited the patient for evaluation. The MET intensivist was also involved in the management if required. From November 2016, we added the implementation of a new real-time AAS to the conventional activation system. With AAS, abnormal vital signs (heart rate, blood pressure, oxygen saturation, and respiratory rate) that met the activation criteria (Table 1) were automatically identified on EMRs and alarms were directly sent to the MET nurses’ phones, as well as a green-highlighted pop-up on the EMR patient list. After receiving the AAS alarm, MET nurses reviewed the indicated patients’ electronic charts and confirmed real RRS activation with a red-highlighted pop-up on the EMR. Other clinicians could notify the RRS of the activated patient with highlight color on the EMR patient list. The two conventional methods of activation were maintained with the AAS.

**Table 1:** Rapid response system criteria of activation and surveillance

Activation criteria
Respiratory
***RR* ≤ *8/min, or* ≥ *28/min***^***a***^
***SpO2* ≤ *90%***^***a***^
Sudden respiratory distressed symptom
Cardiovascular
***HR* ≤ *40/min, or* ≥ *130/min***^***a***^
***SBP* ≤ *80 mmHg, or* ≥ *200 mmHg***^***a***^
SBP 80–90 mmHg with symptoms
Chest pain sustained with nitroglycerin medication
Neurologic
Sudden deterioration of consciousness
Sudden onset facial or extremities paralysis
New onset epilepsy
Non-specific unexplained agitation
Other
Concerns (worries) by physician
Hypo-perfusion signs

Active surveillance criteria
Post-ICU
Higher grade of risk stratification (grade 4 by KPCS-1^b^)
Post-RRS activation

*HR* heart rate, *RR* respiratory rate, *RRS* rapid response system, *SBP* systolic blood pressure, *SpO*_*2*_ blood oxygen saturation

^a^The real-time automatic alerting system (AAS) activation criteria are presented in bold italic text

^b^KPCS-1, the Korean Patient Classification System-1 [23]

### Patient assessment and RRS activation parameters

We identified demographic characteristics (age, sex), primary disease, co-mobidities, severity of conditions, activation date, activation methods, cause of activation, time to response, activation results, and outcome of hospitalization. The severity of patients’ condition was estimated using the Charlson Comorbidity Index (CCI) by Tenth Revision of the International Classification of Diseases (ICD-10) codes [17, 18] and modified early warning score (MEWS) [19]. The activation methods were: (1) a direct phone call from ward doctors and nurses, (2) active screening by MET members, (3) AAS, and (4) code event broadcasting. The main causes of activation were classified as unstable vital signs (heart rate, blood pressure, oxygen saturation, and respiratory rate), chest discomfort, neurologic abnormality, laboratory abnormalities, and “clinician’s concern”. If the patient’s condition did not meet any specific activation criteria, but the clinician was concerned that the patient’s condition would deteriorate, we categorized it as “clinician’s concern.” MET nurses also supported code events and ICU patient transfers from ICU to outside for evaluations and procedures.

In cases of patients with multiple activations, we included all episodes separately if the RRS was activated again after the termination of the previous event. The following activation results were evaluated: (1) the patient was managed by MET nurses or intensivists with consultation alone, (2) the patient was managed at the ward (with further work-up, medications, and transfusion), (3) the patient underwent an unplanned ICU admission, and/or (4) the patient required a consultation for palliative care.

### Outcome measures

The primary outcomes were code rates per 1000 admissions and in-hospital mortality per 1000 admissions from candidate surgical wards during the entire study period. Code events included in-hospital cardiac arrest in the candidates’ surgical wards, and excluded cardiac arrest, which occurred in outpatient clinics, emergency department, ICUs, and procedural areas. Code events were classified as preventable or unpreventable cases, which were decided upon in weekly review consensus meetings by MET members. A preventable code event was an event accompanied by deteriorating signs or symptoms before the arrest; thus, it could be possible to prevent the event if sufficiently managed [9]. The secondary outcomes were the unplanned ICU admission rate per 1000 admissions and related outcomes such as length of ICU stay and mortality in patients with unplanned ICU admission during the study period. Unplanned ICU admission included both activated and non-activated RRS episodes. The length of hospital stay was calculated from the first RRS activation to discharge.

### Statistical analyses

**Fig. 1:**
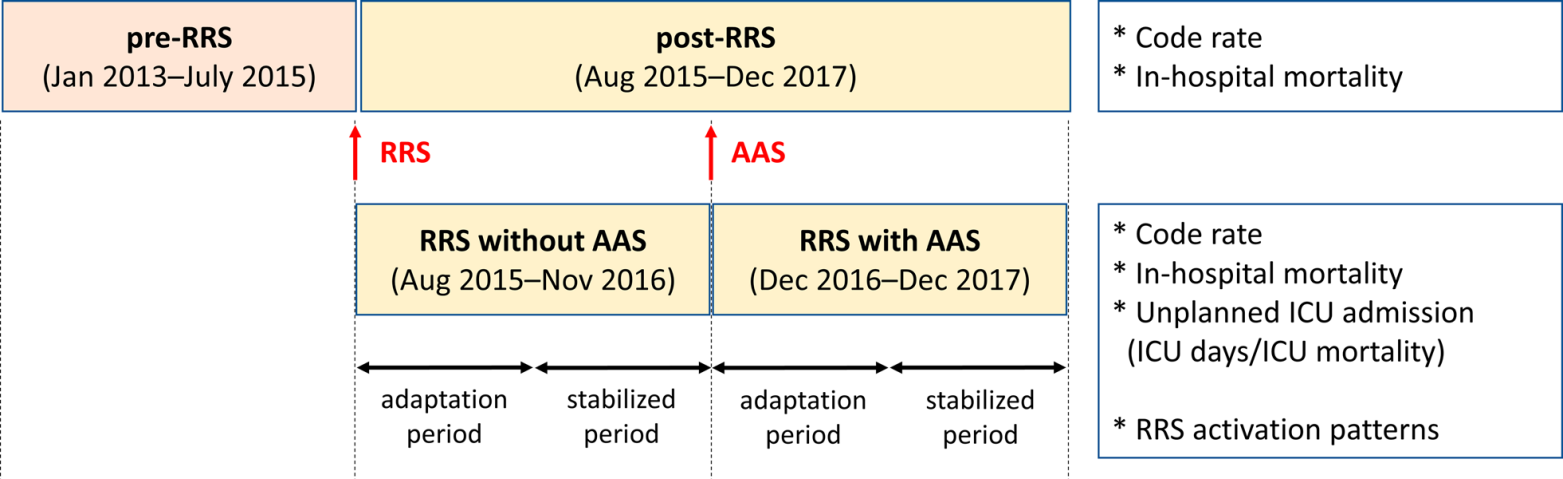
Flow diagram of the study. We analyzed outcomes based on the study period. Patients admitted to the candidate surgical wards during the study period were included, and only patients with RRS activation were included after the implementation of RRS. RRS, rapid response system

We compared outcomes based on periods: pre-RRS (from January 2013 to July 2015) versus post-RRS (from August 2015 to December 2017) and RRS without AAS (from August 2015 to November 2016) versus RRS with AAS (from December 2016 to December 2017). Figure 1 shows the dedicated issues analyzed. We also performed subgroup analyses to evaluate whether there was a difference between the adaptation period (first 6 months after the change of system) and period of stabilization (after 6 months). Based on severity, we sub-analyzed outcomes based on the CCI scores as mild (0–1), moderate (2), or severe (≥ 3), and MEWS as mild (0–1), moderate (2–4), or severe (5–12).

Continuous variables are presented as means and standard deviations for age, the CCI, and MEWS and as median values with interquartile ranges for other variables. Categorical variables are presented as frequencies and proportions. Comparisons between groups were performed using the Pearson and Mantel–Haenszel chi-square test or Fisher’s exact test for categorical variables and analysis of variance or the Kruskal–Wallis test for continuous variables. We performed Cochran–Armitage trend tests to examine time trends by period. *P* values < 0.05 were considered statistically significant.

All analyses were performed using SAS version 9.4, for Windows (SAS Institute, Inc., Cary, NC, USA) and R Statistical Software (version 4.0.0; R Foundation for Statistical Computing, Vienna, Austria).

## Results

### Outcomes of RRS implementation: pre-RRS versus post-RRS

**Fig. 2:**
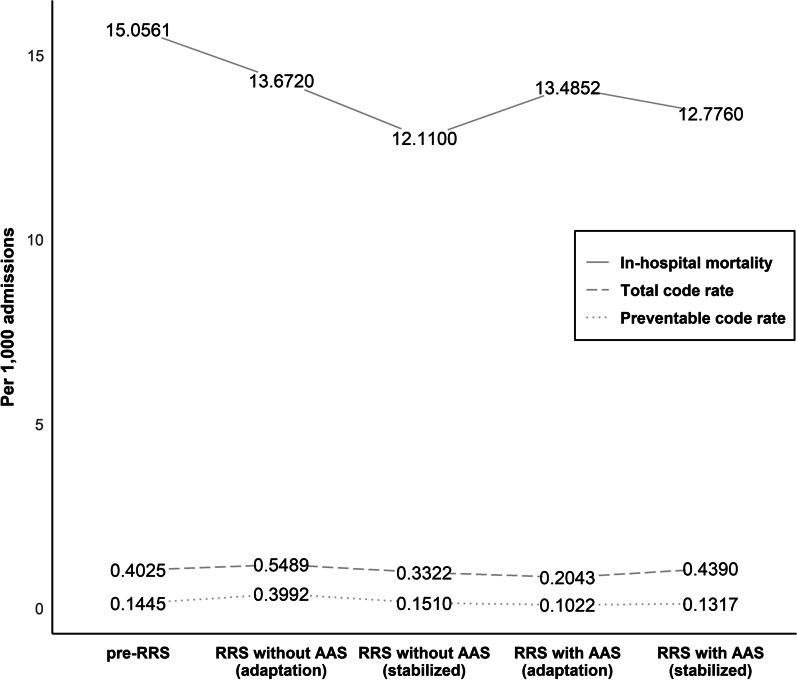
Code rate and unplanned ICU admission rate before and after implementing RRS. ICU, intensive care unit; RRS, rapid response system

During the study period, 192,412 patients were admitted to the surgical wards (96,904 patients during pre-RRS and 95,508 patients during post-RRS). The code rate per 1000 admissions is shown in Fig. 2. There was no difference in the total code rate and preventable code rate between pre-RRS and post-RRS; however, in-hospital mortality per 1000 admissions in the candidate wards decreased significantly from 15.1 to 12.9 after RRS implementation (*p* < 0.0001).

### RRS activation patterns: RRS without AAS versus RRS with AAS

From August 2015 to 2017, the RRS was activated 1883 times (768 times during RRS without AAS and 1115 during RRS with AAS) among 95,508 patients. The baseline characteristics are shown in Table 2. RRS activation per 1000 admissions increased from 14.4 to 26.3 after the introduction of AAS, and severity calculated using the MEWS also increased significantly from 2.5 (± 2.1) to 3.6 (± 2.1) (*p* < 0.001). Most RRS episodes were detected by MET members through manual screening (65.6%) or AAS (44.7%), instead of manual calls from bedside doctors and nurses during the period wherein RRS was activated with/without the AAS. Since AAS was identified based on abnormal vital signs on EMRs, RRS activation owing to unstable vital signs increased from 24.2 to 58.1%. During the RRS without AAS period, RRS was activated in 70.3% of the severe group (MEWS 5–12) by the primary phone call, while in 86.0% of the mild group (MEWS 0–1), RRS was activated by active screening (Additional file 1); however, AAS was the main method of RRS activation (52.3%), instead of the primary phone call (26.3%), in the severe group after AAS implementation. There was no statistical difference in patients’ severity calculated by the CCI using ICD-10 codes (Additional file 2). The code rate and ICU admission were not correlated with the patients’ severity using the CCI; meanwhile, this showed correlation with the MEWS. Consultation and management performed by intensivists decreased, whereas MET nurses’ performance increased significantly from 58.6 to 75.0% after AAS was added to RRS.

**Table 2:** Clinical demographics of RRS activations

Variables	RRS without AAS		RRS with AAS		*p* value^a^
	(n = 768)		(n = 1115)		
	n	(%)	n	(%)	
*RRS activations per 1000 admissions*	14.4		26.3		< 0.001
*Age (year), mean, SD*	63.3 ± 13.7		63.2 ± 16.2		0.905
*Male*	386	(50.2)	622	(55.8)	0.018
*Charlson Comorbidity Index, mean, SD*	2.6 ± 2.5		2.7 ± 2.5		0.1192
Myocardial infarction	6	(0.8)	21	(1.9)	0.0480
Congestive heart failure	17	(2.2)	52	(4.7)	0.0054
Peripheral vascular disorders	50	(6.5)	69	(6.2)	0.7777
Cerebrovascular disease	101	(13.2)	167	(15.0)	0.2649
Dementia	23	(3.0)	45	(4.0)	0.2341
Chronic pulmonary disease	23	(3.0)	50	(4.5)	0.0999
Rheumatic disease	7	(0.9)	24	(2.2)	0.0375
Peptic ulcer disease	23	(3.0)	38	(3.4)	0.6186
Mild liver disease	142	(18.5)	191	(17.1)	0.4473
Diabetes without chronic complication	129	(16.8)	243	(21.8)	0.0074
Diabetes with chronic complication	27	(3.5)	50	(4.5)	0.2969
Hemiplegia or paraplegia	1	(0.1)	7	(0.6)	0.1524
Renal disease	75	(9.8)	149	(13.4)	0.0178
Any malignancy	367	(47.8)	491	(44)	0.1083
Moderate or severe liver disease	21	(2.7)	31	(2.8)	0.9524
Metastatic solid tumor	73	(9.5)	112	(10.0)	0.6990
AIDS/HIV	1	(0.1)	0	(0)	0.4079
*MEWS, mean, SD*	2.5 ± 2.1		3.6 ± 2.1		< 0.001
*Mode of RRS activation*					< 0.001
Call	253	(32.9)	231	(20.7)	
Manual screening	504	(65.6)	373	(33.4)	
AAS	0	(0)	498	(44.7)	
CPR alarm	11	(1.4)	13	(1.2)	
*Person of RRS activation*					< 0.001
Nurse	206	(26.8)	186	(16.7)	
Doctor	47	(6.1)	43	(3.8)	
RRT member (including AAS)	504	(65.6)	871	(78.1)	
Others	11	(1.4)	15	(1.4)	
*Causes of activation (multiple)*					
Respiratory					
Respiratory rate	31	(4.0)	133	(11.9)	< 0.001
Saturation	70	(9.1)	184	(16.5)	< 0.001
Cardiovascular					
Heart rate/arrhythmia	37	(4.8)	205	(18.4)	< 0.001
Blood pressure	40	(5.2)	166	(14.9)	< 0.001
Chest discomfort	11	(1.4)	8	(0.7)	< 0.001
Neurologic	40	(5.2)	19	(1.7)	< 0.001
Others					
Clinicians’ concern	239	(31.1)	255	(22.9)	< 0.001
Abnormal laboratory results	227	(29.6)	95	(8.5)	< 0.001
Education/consultation	58	(7.6)	51	(4.6)	0.0065
Transfer support of ICU patients	26	(3.4)	30	(2.7)	0.3831
Code event	10	(1.3)	8	(0.7)	0.2001
*Time to response (min)*	4 (3–5)		3 (2–5)		0.0842
*Management of activation*					< 0.001
Intensivist + RRT nurse	318	(41.4)	279	(25.0)	
RRT Nurse alone	450	(58.6)	836	(75.0)	
*Results of activation (multiple)*					
ICU transfer	83	(10.8)	114	(10.2)	0.6845
Doctor management	107	(13.9)	63	(5.7)	< 0.001
Doctor consultation	138	(18.0)	132	(11.8)	0.0002
Nurse management	308	(40.1)	705	(63.2)	< 0.001
Nurse consultation/education	155	(20.2)	150	(13.5)	< 0.001
Transfer support	23	(3.0)	26	(2.3)	0.3745
Code event support	9	(1.2)	9	(0.8)	0.4241

Data presented as n (%) or mean (SD). ICU length of stay presented as median (IQR)

*AAS* automatic alerting system, *ICU* intensive care unit, *IQR* interquartile range, *MEWS* modified early warning score, *RRS* rapid response system, *RRT* rapid response team, *SD* standard deviation

^a^*p* value determined using the chi-square test or Fisher’s exact test for categorical variables, Student’s t test for continuous variables

### AAS alert patterns: RRS with AAS

**Fig. 3:**
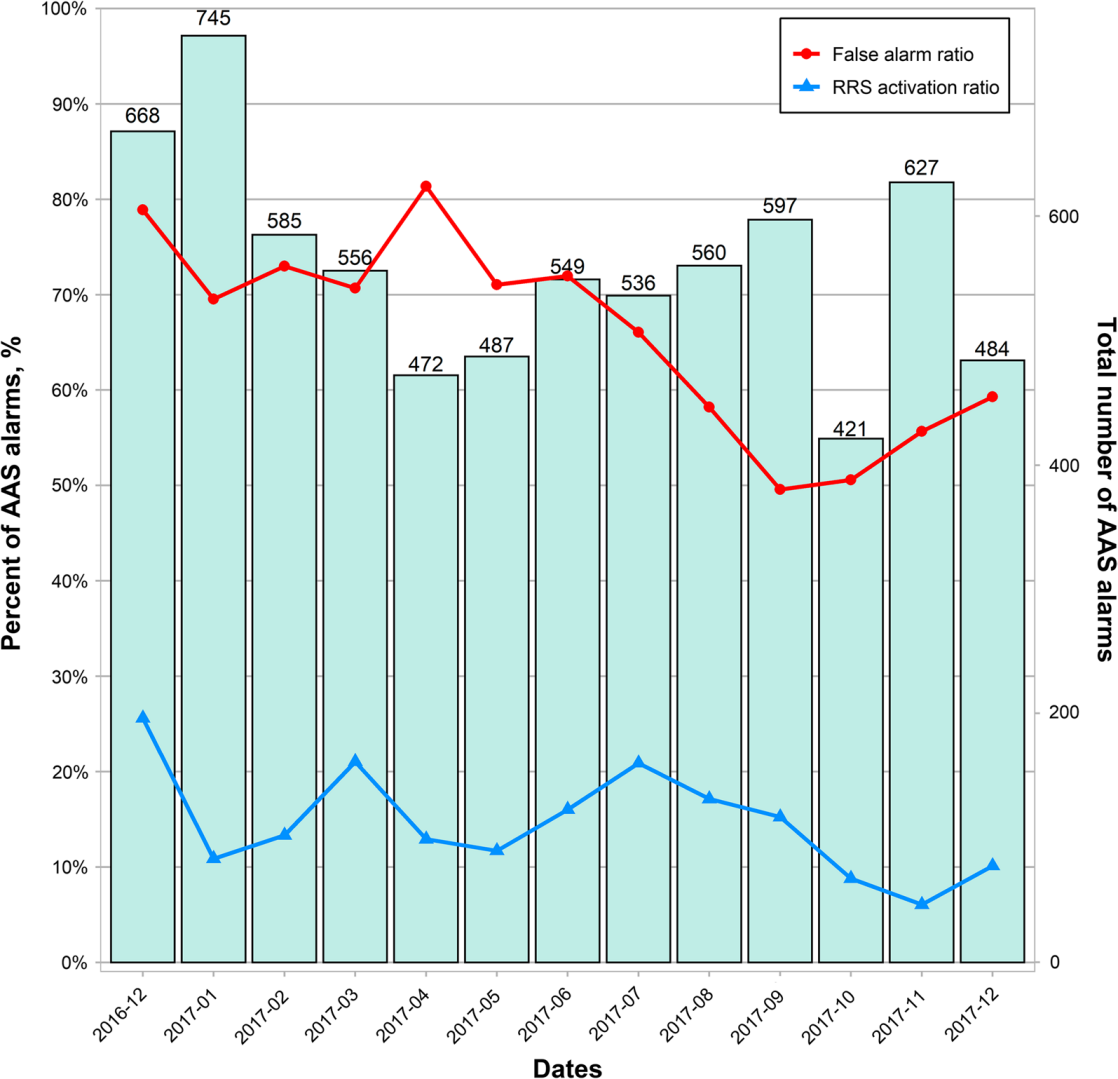
MET activation ratio and false alarm ratio among AAS alarms. MET, medical emergency team; AAS, automatic alerting system

From December 2016 to 2017, AAS alerts were issued 7523 times among 42,354 patients (177.6 times/1000 admissions) during RRS operation time. Among 7523 AAS alerts during the RRS with AAS period, 14.8% (± 5.3%) were activated and managed by RRS. Majority of the false alarms were recording errors (67.0 ± 12.4%) due to human factors during the process of writing down on the electronic charts, although some developed by automatic sensors. The ratio of recording errors among AAS alerts improved with time (78.9% in December 2016 and 59.3% in December 2017) (Fig. 3).

### Outcomes of RRS activation: RRS without AAS and RRS with AAS

We compared the clinical outcomes during periods wherein RRS was activated with/without AAS. There was no difference in total code rates, preventable code rates, and in-hospital mortality rates between the two periods. The unplanned ICU admission rate per 1000 admissions showed no difference between the two periods; however, the proportion of patients admitted to the ICU because of RRS activation increased on addition of AAS (*p* = 0.0005). Among patients who underwent unplanned ICU admissions, the length of ICU stay and ICU mortality improved in patients for whom RRS was activated, while these outcomes were unchanged for patients for whom RRS was not activated during the study period (Table 3). There were no significant changes in clinical outcomes between the adaptation and stabilization periods when either RRS or AAS was introduced. There was no difference in consultation of palliative care between the two periods.

**Table 3:** Code rate and unplanned ICU admission rate before and after the implementation of AAS

Outcomes	RRS without AAS (Aug 2015–Nov 2016)	RRS with AAS (Dec 2016–Nov 2017)	*p* value^a^
Total code rate (per 1000 admissions)	0.4139	0.3305	0.5098
Preventable code rate (per 1000 admissions)	0.2446	0.1181	0.1570
In-hospital mortality (per 1000 admissions)	12.6990	13.1038	0.5814
Unplanned ICU admission (per 1000 admissions)	6.0767	6.5401	0.3678
RRS activation	1.7120	2.7624	0.0005
RRS non-activation	4.3647	3.7777	0.1587
ICU day (median, day, IQR)	2.77 (1.29–6.96)	2.54 (1.13–5.67)	0.0244
RRS activation	2.96 (1.25–8.17)	2.71 (1.04–5.21)	0.0360
RRS non-activation	2.63 (1.29–6.29)	2.54 (1.29–5.79)	0.0917
ICU mortality (N, %)	49 (15.2)	25 (9.0)	0.0225
RRS activation	17 (18.7)	8 (6.8)	0.0092
RRS non-activation	32 (13.8)	17 (10.6)	0.3512
Length of hospital stay from first RRS activation (median, day, IQR)	12.90 (5.27–26.46)	9.33 (4.05–20.13)	0.0956

*AAS* automatic alerting system, *ICU* intensive care unit, *IQR* interquartile range, *RRS* rapid response system

^a^*p* value determined using the chi-square test or Fisher’s exact test for categorical variables and Student’s t test for continuous variables (RRS without AAS vs. RRS with AAS)

## Discussion

The RRS was developed to provide a safety net for deteriorating patients in the process of continuous quality improvement. We analyzed the effect of RRS implementation on the code rate and in-hospital mortality and impact of AAS on the RRS activation pattern and clinical outcomes. The total and preventable code rates per 1000 admissions did not differ between pre-RRS and post-RRS periods; however, there was an improvement in in-hospital mortality after RRS implementation. During the RRS implementation period, the proportion of unplanned ICU admissions with RRS activation was increased. Moreover, the length of ICU stay and ICU mortality rate was significantly lower when AAS was also implemented than when it was not. This was especially true in the RRS-activated group.

This study showed that operation of real-time AAS partly replaced RRS activation by manual alerts from the ward physicians and manual screening by MET members. The conventional manual RRS activation system (without AAS) was limited in identifying unstable patients by MET members if the doctors or nurses in the candidate wards did not call the RRS, despite that the patient’s clinical condition met the RRS activation criteria. Therefore, the deteriorating signs could be: not recognized, recognized but not managed, or be managed and delayed, or insufficiently managed. MET nurses had to spend more time screening EMR data to identify “at risk” patients; however, with AAS, all patients who had abnormal vital signs on the EMR and met the AAS criteria were automatically identified, and MET members could easily identify unstable patients in real time.

With the initiation of AAS, the activation rate also significantly increased from 14.4 times to 26.3 times per 1000 admissions. In addition, our study showed a shortened ICU stay and improved ICU mortality in unplanned ICU admissions after AAS was introduced, despite the increased severity calculated using the MEWS. While there is no clear cut-off for defining the ideal number of RRS activations, studies show that higher activation rate contributes to improvement of patient outcomes. Jones et al. [20] reported that increasing the MET activations was associated with reduction in cardiac arrests. Through a cluster randomized controlled trial, Chen et al. [10] showed that for every 10% increase in the proportion of MET alerts, unexpected cardiac arrests, overall cardiac arrests, and unexpected deaths reduced by 2, 2.2, and 0.94 times, respectively. In our study, improvements in clinical outcomes after AAS implementation were only observed in ICU patients for whom RRS was activated. Additionally, the RRS-activated group showed higher MEWS scores than did those without RRS activation. Therefore, we infer that RRS activation contributed to improved outcomes.

To evaluate the learning period effect, we compared the outcomes between the adaptation and stabilization periods of RRS and AAS implementation. The in-hospital mortality tended to improve, although not significantly, in the stabilization period than in the adaptation period with both RRS and AAS implementation (Fig. 2). The response to RRS was initially physician-led; however, with time, the use of RRS stabilized in the hospital and the response became nurse-led with the MET showing increased performance of nurses and a consultation-based role of physicians. Few studies exist reporting automatic alarm incidence and proportion of RRS activation. In this study, 7523 AAS alerts were issued and 14.8% (± 5.3%) were activated and managed by RRS during the AAS period. Because of the need to endure excessive AAS alarms and with the increasing sensitivity to detect deteriorating patients in RRS, alarm fatigue may results from the frequent AAS alarms and recording errors when operating AAS based on EMR, especially during the adaptation period. Because most false alarm were recording errors by human, we appraised the process of RRS with AAS and fed-back to the ward clinicians to reduce the incidence of errors. The ratio of recording errors among AAS alarms improved from 78.9% in November 2016 to 59.3% in December 2017 (Fig. 3). All these efforts are a continuous process of quality improvement. This study had some limitations. First, in this single-center study, which included patients who were admitted in the surgical wards, the analysis was performed retrospectively, although the data were collected prospectively. Therefore, our results may not be generalizable to other hospitals or departments. Second, although little is known regarding the influence of RRS operating time on clinical outcomes [16, 21], limited RRS operation time in this study could be a limitation; our RRS was only operated from 7 AM to 7 PM by intensivists during weekdays. However, our study showed improved clinical outcomes even with the restricted RRS operating duration. Third, because our study only included 16 months of the pre-AAS and 13 months of the post-AAS periods, we could not consider seasonal change and time bias. Fourth, we could not identify all patients for whom RRS was not activated but only those admitted to candidate surgical wards during the study period. In addition, it is practically impossible to exclude the Hawthorne effect, which may have arose from a behavioral change due to awareness of being participating in the AAS, on our clinical outcomes [22]. Despite these limitations, this study demonstrates two practice patterns of RRS and the difference in each system.

## Conclusions

In conclusion, we found an improvement in in-hospital mortality after RRS implementation. Real-time AAS based on EMR enabled easy identification of deteriorating patients and increased MET alerts. Early detection and intervention by activation of RRS with AAS may improve the ICU mortality rate and shorten ICU duration, despite the higher MEWSs than those for whom RRS was not activated.

## Supplementary Information

**Additional file 1:** Outcomes of the rapid response system activations based on the modified early warning score.

**Additional file 2:** Outcomes of the rapid response system activations based on the Charlson commodify index.

## Data Availability

The datasets used and/or analyzed during the current study are available from the corresponding author after permission of the Institutional Review Board of Seoul National University Hospital on reasonable request.

## Abbreviations

AAS: Automatic alerting system
CCI: Charlson Comorbidity Index
EMR: Electronic medical records;
ICU: Intensive care unit
MET: Medical emergency team
MEWS: Modified early warning score
RRS: Rapid response system
