# Supplementary material for: Incorporating a real-time automatic alerting system based on electronic medical records could improve rapid response systems: a retrospective cohort study
Source: Scand J Trauma Resusc Emerg Med. 2021 Dec 4;29:164. doi: 10.1186/s13049-021-00979-y (PMC8643026; doi:10.1186/s13049-021-00979-y)
Supplement: Supplementary file 1 — Additional file 1: Outcomes of the rapid response system activations based on the modified early warning score. [file 13049_2021_979_MOESM1_ESM.docx]

**Additional File**

**Additional File 1.** Outcomes of the rapid response system activations based on the modified early warning score

| **Variables** | | **MEWS**  **0-1** | | **MEWS**  **2-4** | | **MEWS**  **5-12** | | **p-value**^a^ |
| --- | --- | --- | --- | --- | --- | --- | --- | --- |
|  |  | **w/o AAS**  **(n=328,**  **42.7%)** | **w/ AAS**  **(n=222, 19.9%)** | **w/o AAS**  **(n=318, 41.4%)** | **w/ AAS**  **(n=569, 51.0%)** | **w/o AAS**  **(n=111, 14.5%)** | **w/ AAS**  **(n=323, 30.0%)** |  |
| Age (years), mean, SD | | 62.4 ± 12.8 | 64.0 ± 16.2 | 63.8 ± 14.6 | 63.2 ± 16.1 | 63.4 ± 14.1 | 62.8 ± 16.1 | 0.8049 |
| Male | | 167 (50.9) | 125 (56.3) | 151 (47.5) | 310 (54.5) | 62 (55.9) | 186 (57.6) | 0.0253 |
| MEWS, mean, SD | | 1.0 ± 0.1 | 1.0 ± 0.1 | 2.7 ± 0.8 | 3.1 ± 0.8 | 6.6 ± 2.0 | 6.2 ± 1.6 | <0.001 |
| Mode of activation | |  |  |  |  |  |  | <0.001 |
|  | Call | 44 (13.4) | 36 (16.2) | 120 (37.7) | 109 (19.2) | 78 (70.3) | 85 (26.3) |  |
|  | Screening | 282 (86.0) | 122 (55.0) | 197 (62.0) | 192 (33.7) | 25 (22.5) | 59 (18.3) |  |
|  | AAS | 0 (0) | 63 (28.4) | 0 (0) | 266 (46.8) | 0 (0) | 169 (52.3) |  |
|  | CPR alarm | 2 (0.6) | 1 (0.5) | 1 (0.3) | 2 (0.3) | 8 (7.2) | 10 (3.1) |  |
| Person of activation | |  |  |  |  |  |  | <0.001 |
|  | Nurse | 36 (11.0) | 30 (13.5) | 97 (30.5) | 90 (15.8) | 62 (55.9) | 66 (20.4) |  |
|  | Doctor | 8 (2.4) | 6 (2.7) | 23 (7.2) | 18 (3.2) | 16 (14.4) | 18 (5.6) |  |
|  | RRT member | 282 (86.0) | 185 (83.3) | 197 (62.0) | 458 (80.5) | 25 (22.5) | 228 (70.6) |  |
|  | Others | 2 (0.6) | 1 (0.5) | 1 (0.3) | 3 (0.5) | 8 (7.2) | 11 (3.4) |  |
| Causes of activations (multiple) | |  |  |  |  |  |  |  |
|  | Respiratory rate | 1 (0.3) | 3 (1.4) | 15 (4.7) | 58 (10.2) | 15 (13.5) | 72 (22.3) | 0.0003 |
|  | Saturation | 16 (4.9) | 58 (26.1) | 35 (11.0) | 84 (14.8) | 19 (17.1) | 42 (13.0) | <0.001 |
|  | Heart rate/arrhythmia | 4 (1.2) | 4 (1.8) | 8 (2.5) | 89 (15.6) | 25 (22.5) | 112 (34,7) | <0.001 |
|  | Blood pressure | 4 (1.2) | 2 (0.9) | 24 (7.6) | 106 (18.6) | 12 (10.8) | 58 (18.0) | <0.001 |
|  | Chest discomfort | 8 (2.4) | 1 (0.5) | 3 (0.9) | 7 (1.2) | 0 (0) | 0 (0) | 0.3296 |
|  | Neurology | 10 (3.1) | 2 (0.9) | 18 (5.7) | 8 (1.4) | 12 (10.8) | 9 (2.8) | <0.001 |
|  | Clinicians’ concerns | 114 (34.8) | 75 (33.8) | 103 (32.4) | 150 (26.4) | 22 (19.8) | 30 (9.3) | 0.0129 |
|  | Abnormal laboratory results | 151 (46.0) | 53 (23.9) | 74 (23.3) | 31 (5.5) | 2 (1.8) | 11 (3.4) | <0.001 |
|  | Education/consultation | 19 (5.8) | 14 (6.3) | 32 (10.1) | 23 (4.0) | 7 (6.3) | 14 (4.3) | 0.0060 |
|  | Transfer support | 4 (1.2) | 4 (1.8) | 15 (4.7) | 11 (1.9) | 7 (6.3) | 14 (4.3) | 0.0542 |
|  | Code event | 1 (0.3) | 0 | 1 (0.3) | 0 (0) | 8 (7.2) | 8 (2.5) | 0.0073 |
|  | Others | 5 (1.5) | 12 (5.4) | 14 (4.4) | 39 (6.8) | 4 (3.6) | 11 (3.4) | 0.0194 |
| Time to response (min) | | 5 (3.5-5) | 4 (2-5) | 4 (2.5-5) | 3 (3-5) | 4 (3-5) | 3 (2-5) | 0.1962 |
| Management of activation | |  |  |  |  |  |  | <0.001 |
|  | Intensivist + RRT nurse | 117 (35.7) | 44 (19.8) | 140 (44.0) | 126 (22.1) | 61 (55.0) | 109 (33.8) |  |
|  | RRT Nurse only | 211 (64.3) | 178 (80.2) | 178 (56.0) | 443 (77.9) | 50 (45.0) | 214 (66.2) |  |
| Results of activation | |  |  |  |  |  |  |  |
|  | ICU transfer | 10 (3.0) | 6 (2.7) | 33 (10.4) | 42 (7.4) | 40 (36.0) | 66 (20.4) | 0.0010 |
|  | Doctor management | 27 (8.2) | 4 (1.8) | 60 (18.9) | 33 (5.8) | 20 (18.0) | 26 (8.0) | <0.001 |
|  | Doctor consultation | 81 (24.7) | 35 (15.8) | 50 (15.7) | 63 (11.1) | 7 (6.3) | 34 (10.5) | 0.0133 |
|  | Nurse management | 154 (47.0) | 154 (69.4) | 127 (39.9) | 388 (68.2) | 27 (24.3) | 163 (50.5) | <0.001 |
|  | Consultation/education | 75 (22.9) | 45 (20.3) | 57 (17.9) | 75 (13.2) | 12 (10.8) | 30 (9.3) | 0.0542 |
|  | Transfer support | 4 (1.2) | 3 (1.4) | 13 (4.1) | 9 (1.6) | 6 (5.4) | 13 (4.0) | 0.0554 |
|  | Code event support | 0 (0) | 0 (0) | 0 (0) | 0 (0) | 9 (8.1) | 9 (2.8) | 0.0154 |
| 30-day mortality | | 7 (2.1) | 14 (6.3) | 46 (14.5) | 56 (9.8) | 25 (22.5) | 73 (22.6) | 0.5440 |

Data presented as n (%) or mean (SD). ICU length of stay presented as median (IQR)

CPR, cardiopulmonary resuscitation; ICU, intensive care unit; IQR, interquartile range; SD: standard deviation

^a^p-value by Cochran–Mantel–Haenszel test for categorical variables, by analysis of variance (ANOVA) for age, and by Kruskal–Wallis test for other continuous variables
