# Supplementary material for: Incorporating a real-time automatic alerting system based on electronic medical records could improve rapid response systems: a retrospective cohort study
Source: Scand J Trauma Resusc Emerg Med. 2021 Dec 4;29:164. doi: 10.1186/s13049-021-00979-y (PMC8643026; doi:10.1186/s13049-021-00979-y)
Supplement: Supplementary file 2 — Additional file 2: Outcomes of the rapid response system activations based on the Charlson commodify index. [file 13049_2021_979_MOESM2_ESM.docx]

**Additional File**

**Additional File 2.** Outcomes of the rapid response system activations based on the Charlson Comorbidity Index

| **Variables** | | **CCI score**  **0-1** | | **CCI score**  **2** | | **CCI score**  **≥3** | | **p-value**^a^ |
| --- | --- | --- | --- | --- | --- | --- | --- | --- |
|  |  | **w/o AAS**  **(n=242, 31.5%)** | **w/ AAS**  **(n=341, 30.6%)** | **w/o AAS**  **(n=269, 35.0%)** | **w/ AAS**  **(n=346, 31.0%)** | **w/o AAS**  **(n=257, 33.5%)** | **w/ AAS**  **(n=428, 38.4%)** |  |
| Age (years), mean, SD | | 62.8 ± 15.7 | 62.2 ± 19.2 | 63.1 ± 11.8 | 59.6 ± 14.7 | 64.0 ± 13.7 | 67.0 ± 13.7 | <0.001 |
| Male | | 115 (47.5) | 163 (47.8) | 140 (52.0) | 198 (57.2) | 131 (51.0) | 261 (61.0) | 0.0218 |
| MEWS, mean, SD | | 2.8 ± 2.3 | 3.5 ± 2.0 | 2.5 ± 2.0 | 3.7 ± 2.1 | 2.4 ± 2.0 | 3.5 ± 2.2 | <0.001 |
| Mode of activation | |  |  |  |  |  |  | <0.001 |
|  | Call | 106 (43.8) | 81 (23.8) | 69 (25.7) | 60 (17.3) | 78 (30.4) | 90 (21.0) |  |
|  | Screening | 131 (54.1) | 105 (30.8) | 196 (72.9) | 110 (31.8) | 177 (68.9) | 158 (36.9) |  |
|  | AAS | 0 (0) | 152 (44.6) | 0 (0) | 171 (49.4) | 0 (0) | 175 (40.9) |  |
|  | CPR alarm | 5 (2.1) | 3 (0.9) | 4 (1.5) | 5 (1.4) | 2 (0.8) | 5 (1.2) |  |
| Person of activation | |  |  |  |  |  |  | <0.001 |
|  | Nurse | 85 (35.1) | 66 (19.4) | 57 (21.2) | 44 (12.7) | 64 (24.9) | 26 (17.8) |  |
|  | Doctor | 21 (8.7) | 15 (4.4) | 12 (4.5) | 14 (4.0) | 14 (5.4) | 14 (3.3) |  |
|  | RRT member | 131 (54.1) | 257 (75.4) | 196 (72.9) | 281 (81.2) | 177 (68.9) | 333 (77.8) |  |
|  | Others | 5 (2.1) | 3 (0.9) | 4 (1.5) | 7 (2.0) | 2 (0.8) | 5 (1.2) |  |
| Causes of activations (multiple) | |  |  |  |  |  |  |  |
|  | Respiratory rate | 7 (2.9) | 44 (12.9) | 10 (3.7) | 40 (11.6) | 14 (5.4) | 49 (11.4) | <0.001 |
|  | Saturation | 23 (9.5) | 53 (15.5) | 25 (9.3) | 54 (15.6) | 22 (8.6) | 77 (18.0) | <0.001 |
|  | Heart rate/arrhythmia | 14 (5.8) | 72 (21.1) | 13 (4.8) | 67 (19.4) | 10 (3.9) | 65 (15.2) | <0.001 |
|  | Blood pressure | 19 (7.9) | 46 (13.5) | 5 (1.9) | 57 (16.5) | 16 (6.2) | 63 (14.7) | <0.001 |
|  | Chest discomfort | 4 (1.7) | 3 (0.9) | 6 (2.2) | 4 (1.2) | 1 (0.4) | 1 (0.2) | 0.1650 |
|  | Neurology | 18 (7.4) | 6 (1.8) | 12 (4.5) | 6 (1.7) | 10 (3.9) | 7 (1.6) | <0.001 |
|  | Clinicians’ concerns | 87 (36.0) | 74 (21.7) | 81 (30.1) | 77 (22.3) | 71 (27.6) | 104 (24.3) | <0.001 |
|  | Abnormal laboratory results | 38 (15.7) | 12 (3.5) | 93 (34.6) | 29 (8.4) | 96 (37.4) | 54 (12.6) | <0.001 |
|  | Education/consultation | 18 (7.4) | 19 (5.6) | 22 (8.2) | 19 (5.5) | 18 (7.0) | 13 (3.0) | 0.0086 |
|  | Transfer support | 10 (4.1) | 10 (2.9) | 9 (3.3) | 12 (3.5) | 7 (2.7) | 8 (1.9) | 0.4261 |
|  | Code event | 5 (2.1) | 2 (0.6) | 3 (1.1) | 2 (0.6) | 2 (0.8) | 4 (0.9) | 0.2002 |
|  | Others | 17 (7.0) | 28 (8.2) | 9 (3.3) | 11 (3.2) | 8 (3.1) | 24 (5.6) | 0.2471 |
| Time to response (min) | | 4 (2-5) | 3 (2-5) | 4 (3-5) | 3 (3-5) | 4 (3-5) | 3 (2-5) | 0.2646 |
| Management of activation | |  |  |  |  |  |  | <0.001 |
|  | Intensivist + RRT nurse | 107 (44.2) | 85 (24.9) | 106 (39.4) | 826 (23.7) | 105 (40.9) | 112 (26.2) |  |
|  | RRT Nurse only | 135 (55.8) | 256 (75.1) | 163 (60.6) | 264 (76.3) | 152 (59.1) | 316 (73.8) |  |
| Results of activation | |  |  |  |  |  |  |  |
|  | ICU transfer | 23 (9.5) | 37 (10.9) | 30 (11.2) | 35 (10.1) | 30 (11.7) | 42 (9.8) | 0.6845 |
|  | Doctor management | 44 (18.2) | 22 (6.5) | 32 (11.9) | 16 (4.6) | 31 (12.1) | 25 (5.8) | <0.001 |
|  | Doctor consultation | 46 (19.0) | 32 (9.4) | 45 (16.7) | 40 (11.6) | 47 (18.3) | 60 (14.0) | <0.001 |
|  | Nurse management | 76 (31.4) | 220 (64.5) | 125 (46.5) | 210 (60.7) | 107 (41.6) | 275 (64.3) | <0.001 |
|  | Consultation/education | 56 (23.1) | 47 (13.8) | 49 (18.2) | 58 (16.8) | 50 (19.5) | 45 (10.5) | <0.001 |
|  | Transfer support | 9 (3.7) | 8 (2.3) | 8 (3.0) | 11 (3.2) | 6 (2.3) | 7 (1.6) | 0.4174 |
|  | Code event support | 5 (2.1) | 2 (0.6) | 2 (0.7) | 2 (0.6) | 2 (0.8) | 5 (1.2) | 0.4066 |
| 30-day mortality | | 24 (9.9) | 23 (6.7) | 24 (8.9) | 44 (12.7) | 32 (12.5) | 76 (17.8) | 0.0021 |

Data presented as n (%) or mean (SD). ICU length of stay presented as median (IQR)

CPR, cardiopulmonary resuscitation; ICU, intensive care unit; IQR, interquartile range; SD: standard deviation

^a^p-value by Cochran–Mantel–Haenszel test for categorical variables, by analysis of variance (ANOVA) for age, and by Kruskal–Wallis test for other continuous variables
